# Near-Infrared Spectroscopy as a Potential COVID-19 Early Detection Method: A Review and Future Perspective

**DOI:** 10.3390/s22124391

**Published:** 2022-06-10

**Authors:** Muna E. Raypah, Asma Nadia Faris, Mawaddah Mohd Azlan, Nik Yusnoraini Yusof, Fariza Hanim Suhailin, Rafidah Hanim Shueb, Irneza Ismail, Fatin Hamimi Mustafa

**Affiliations:** 1 School of Physics, Universiti Sains Malaysia, George Town 11800, Pulau Pinang, Malaysia; muna_ezzi@usm.my; 2 Institute for Research in Molecular Medicine (INFORMM), Universiti Sains Malaysia Health Campus, Kubang Kerian 16150, Kelantan, Malaysia; nadiasolehah@student.usm.my (A.N.F.); mawaddahmohdazlan@usm.my (M.M.A.); nikyus@usm.my (N.Y.Y.); hanimkk@usm.my (R.H.S.); 3 Department of Physics, Faculty of Science, Universiti Teknologi Malaysia, Skudai 81310, Johor, Malaysia; farizahanim@utm.my; 4 Department of Medical Microbiology and Parasitology, School of Medical Sciences, Universiti Sains Malaysia, Health Campus, Kubang Kerian 16150, Kelantan, Malaysia; 5 Advanced Devices & System (ADS) Research Group, Department of Electrical & Electronic Engineering, Faculty of Engineering and Built Environment, Universiti Sains Islam Malaysia, Bandar Baru Nilai, Nilai 71800, Negeri Sembilan, Malaysia

**Keywords:** near-infrared spectroscopy, SARS-CoV-2, COVID-19, viruses, chemometrics, diagnostics

## Abstract

The coronavirus disease 2019 (COVID-19) pandemic is a worldwide health anxiety. The rapid dispersion of the infection globally results in unparalleled economic, social, and health impacts. The pathogen that causes COVID-19 is known as a severe acute respiratory syndrome coronavirus 2 (SARS-CoV-2). A fast and low-cost diagnosis method for COVID-19 disease can play an important role in controlling its proliferation. Near-infrared spectroscopy (NIRS) is a quick, non-destructive, non-invasive, and inexpensive technique for profiling the chemical and physical structures of a wide range of samples. Furthermore, the NIRS has the advantage of incorporating the internet of things (IoT) application for the effective control and treatment of the disease. In recent years, a significant advancement in instrumentation and spectral analysis methods has resulted in a remarkable impact on the NIRS applications, especially in the medical discipline. To date, NIRS has been applied as a technique for detecting various viruses including zika (ZIKV), chikungunya (CHIKV), influenza, hepatitis C, dengue (DENV), and human immunodeficiency (HIV). This review aims to outline some historical and contemporary applications of NIRS in virology and its merit as a novel diagnostic technique for SARS-CoV-2.

## 1. Introduction

On 12 March 2020, the World Health Organization (WHO) declared the coronavirus disease 2019 (COVID-2019) as a public health emergency of international concern, with rising reported cases worldwide of up to 20,000 [1,2]. To date, the rapid upsurge of confirmed COVID-19 cases is still uncontrollable. Nowadays, many countries impose a lockdown where individuals are instructed to bide at home and movement is restricted in order to break the chain of infection. Since no effective treatments are provided for COVID-19, the number of deaths globally is anticipated to rise [3,4]. The available vaccines have created some degree of confidence and many countries have started administering them [5]. However, developing countries cannot afford to purchase expensive vaccines [6], which partly contribute to high cases and fatal infections. Early diagnosis, isolation, and treatment are important to manage the infection and control the pandemic [7]. A technique for discriminating the virus with a high specificity and sensitivity [8] and an advancement in the molecular apparatuses for accurate diagnosis of SARS-CoV-2 infections are in high demand [9]. This is because the clinical symptoms of COVID-19 including fever, dry cough, headache, muscle pain, and tiredness [10,11] are identical to other respiratory viral infections such as influenza [11,12]. Thus, an accurate diagnostic method for differentiating those infected with SARS-CoV-2 from healthy individuals or other viruses is crucial for prompt and effective disease control and treatment.

Presently, techniques with various principles, charges, and sensitivities have been reported for COVID-19 diagnosis [13]. The standard diagnostic methods that are being employed for detecting viral infections include enzyme-linked immunosorbent assay (ELISA), conventional or real-time reverse transcription-polymerase chain reaction (RT-PCR), immunofluorescence assay (IFA), and Western blotting (WB) [14]. The detection of SARS-CoV-2 RNA or antibody-induced following infection is the common approach used to confirm the disease. The viral RNA is usually identified using RT-PCR or nucleic acid hybridization techniques and viral antibody/antigen is detected via immunological and serological analyses such as ELISA. However, none of the aforementioned methods are ideal in terms of cost, rapidity, precision, and large-scale regular screening [15]. The massive number of SARS-CoV-2-infected individuals and the reagent requirements of the current methods need alternative diagnostic tools.

Following the improvement in the miniaturization of the devices that allow highly reproducible spectral measurements, optical-based tools are now becoming progressively prevalent in medical science, specifically for virus detection [16,17,18]. Optical methods such as optical sensing, spectroscopy, and imaging established a great potential [19]. At present, various review articles, research, laboratories, and companies dedicated their attention to applying the optical techniques for sensing the COVID-19 virus [8,19,20,21]. The near-infrared spectroscopy (NIRS) technology has shown huge advantages and potential as a diagnostic method in offering physical and chemical information [22], and also, details on the molecular compositions and structures of biological components [23]. The symptoms of the virus infection affect the molecular compositions that result in observing a specific signature in the spectral characteristics of the infected samples that are used for the illness diagnosis. NIRS has been employed successfully for detecting viruses in humans such as hepatitis C, HIV, and influenza [24,25,26,27,28]. The ability of the NIRS to distinguish between normal and virus-infected specimens in a short time with good accuracy, suggests that NIRS is a promising technology in virology [15,25,29,30]. In this review, the applications of the NIRS as a mature technology for diagnosing different types of viruses are presented and its great potential to detect the SARS-CoV-2 disease is highlighted. A comprehensive summary of the applications of the NIRS as a diagnostic method in the virological field is also provided. This study may help researchers and clinicians to develop proper approaches for the timely and effective detection of SARS-CoV-2 using the NIRS technique.

## 2. Structural Properties of Coronavirus and Relation to COVID-19 Diagnosis

**Figure 1:**
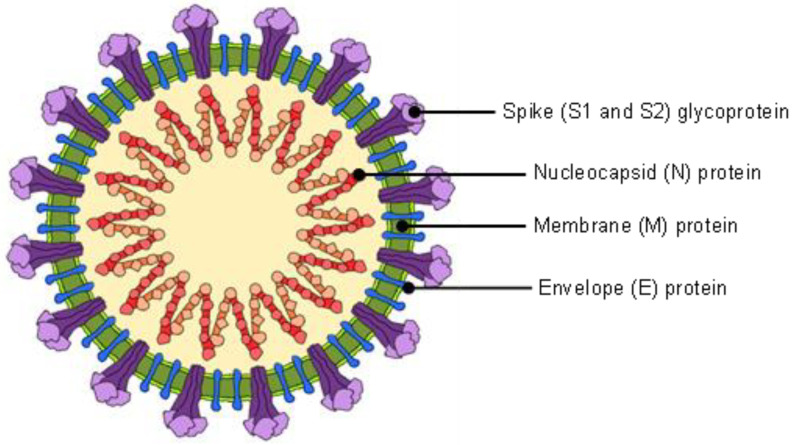
SARS-CoV-2 structure.

Coronavirus affects humans, mammals, and avian groups and causes respiratory tract infections. It ranges from moderate conditions such as the common cold to lethal conditions, which are known as Severe Acute Respiratory Syndrome (SARS), Middle East Respiratory Syndrome (MERS), and COVID-19 (SARS-CoV-2). The SARS-CoV-2 virus encodes four structural proteins of spike glycoprotein (S), membrane protein (M), envelope protein (E), and nucleocapsid protein (N) [31]. Figure 1 shows the composition of the SARS-CoV-2. N-protein that is located in the center of the SARS-CoV-2 virus (N-protein also at the center for other viruses) contains information that can be used for diagnostics and detection. The gold standard method of polymerase chain reaction (PCR) or its advancement method of RT-qPCR uses ribonucleic acid (RNA) samples to diagnose COVID-19, where the RNA is one of the substances in the N-protein. The N-protein is also involved in antigen detection, where the whole virus of SARS-CoV-2 is used as the interest sample [32]. The available methods for antigen detection include lateral flow assay (antigen rapid test kit) and ELISA [33,34].

## 3. Background on Near-Infrared Spectroscopy

**Figure 2:**
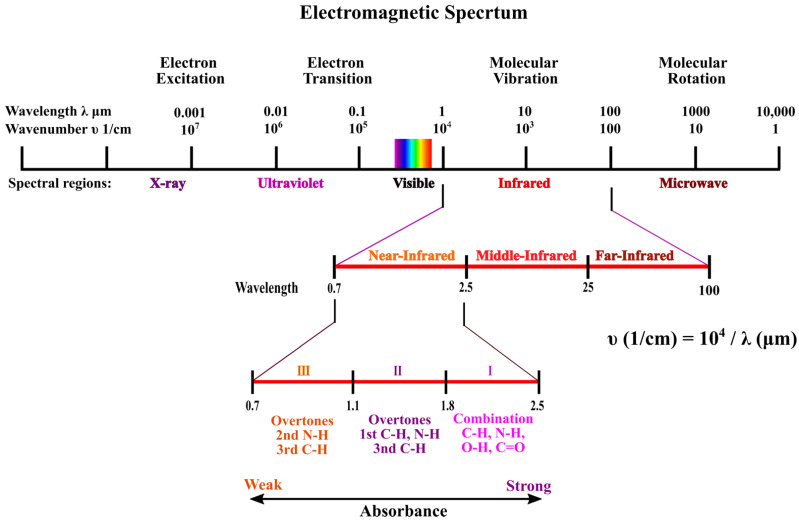
Electromagnetic spectrum highlighting IR and NIR regions.

The discovery of NIR radiation in the 1800s by was made William Herschel. It was started when Herschel isolated the electromagnetic (EM) spectrum using a prism and found that the temperature markedly increased in the ‘direction of’ and beyond the red region (visible region). The NIR region lies between the visible and microwave region in the electromagnetic spectrum at the wavelength range of 750–2500 nm (13,333–4000 cm^−1^) as shown in Figure 2.

The interaction of EM radiation with matter in the NIR area can be manipulated for sensing or detection determinations [35,36]. Light transmission through any material is dependent on reflection, absorption, and scattering [36]. When an NIR light is incident on a particular material, the light is either absorbed or scattered [37]. These absorptions and scatterings can dampen the NIR light intensity, which strongly depends on the composition of the material itself. Light scattering is also controlled by the refractive index of the material [38]. These optical properties of a material are expressed using the following terms: absorption coefficient, $\mu_{a}\;$(${\mathrm{mm}}^{-1}$), scattering coefficient, $\mu_{s}\;$(${\mathrm{mm}}^{-1}$), and refractive index, n [39]. The measurement of optical properties is important for the proper design and manufacture of devices as well as elucidating diagnostic testing [40]. The optical properties can also be utilized to study light propagation in the material using a light transport model, as well as to determine optimal wavelengths and environmental effects.

**Figure 3:**
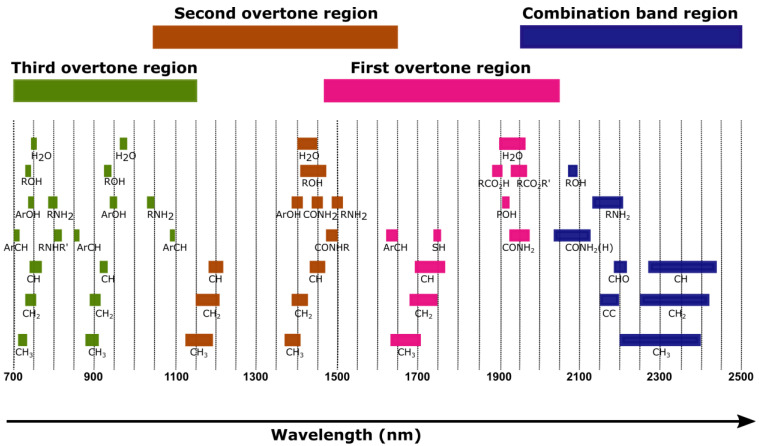
Overtones and combinations of NIR band assignments.

During the interaction between the NIR radiation and a material of interest, the molecular bonds of O-H, C-H, N-H, and C=O in the materials molecular structure experience vibrations due to the absorption of the radiation. The overtones and combinations of NIR band assignments are shown in Figure 3. A higher overtone indicates a higher energy requirement for the molecular bonds to vibrate. After irradiation with the NIR light, the electrons in the molecules are sufficiently energized to move from the ground state to excited vibrational states either on the first, second, or third overtones or levels of energy [41]. At this state, the rate of absorption relies on the wavelength and chemical composition of the material (number of O-H, C-H, N-H, and C=O) because the fundamental vibrations of the chemical bonds are associated with atomic interactions with NIR light using an energy equation of *E = hν* (*h* is Planck’s constant and *ν* is the frequency) [42]. The NIR absorption spectra with overtone regions indicate the dominant absorption of NIR light due to the molecular bonds of functional groups present at the specific wavelengths, thus explaining the unique absorption spectra exhibited by the material.

The optical properties of the absorption coefficient $\mu_{a}$ is known as the portion of light absorbed (A) divided by the thickness (d) of a thin layer or concentration of material [43,44,45]. A high coefficient ($\mu_{a}$) value indicates that the sample experiences more absorption of light per thickness or concentration. The Beer–Lambert law is appropriate for the determination of the concentration of samples, which can be formulated as:

(1) $$A=\log_{10}\left(\frac{I_{o}}{I}\right)=\alpha .C.d$$

where light attenuation is measured in optical density (OD) and A, $I_{o}$, I, α, C, and denote attenuation (the sample absorbance at wavelength, λ), the incident light intensity, the transmitted light intensity, the proportionality constant (referred to as the molar extinction coefficient (L/mol·cm)), the concentration of samples (mol/L), and the optical path length (cm), respectively. Based on the Beer–Lambert law, there is a direct proportionality between light attenuation and sample thickness as well as the concentration of compounds in the sample. The optical path length is dependent on the subjects, the size of containers to place the samples, the measured region, and the wavelength. The Beer–Lambert law, however, is only valid for liquids and gases materials within certain thickness and concentration ranges.

The scattering coefficient, $\mu_{s}$ is defined as the probability of a light being scattered in a material per path length. The $\mu_{s}$ is directly related to the refractive index, n, and particle sizes because strong scattering occurs in two conditions. Firstly, when the incident light wavelength is similar to the particle size [46]. The collision between the light and particles create Mie scattering, either forwards or backwards. Secondly, when the incident light meets a boundary with a mismatched refractive index, *n*. Alterations in the light speed at the interface or boundary separating surfaces with different *n* values can lead to high scattering. The *n* is also a measurement parameter of optical density, where the light transmits faster in low optical density materials or low concentration solutions. Light scattering spectroscopy is used in sensing applications to determine the physical structure and size of molecules [47].

### 3.1. Instrumentation and Sample Presentation

**Figure 4:**
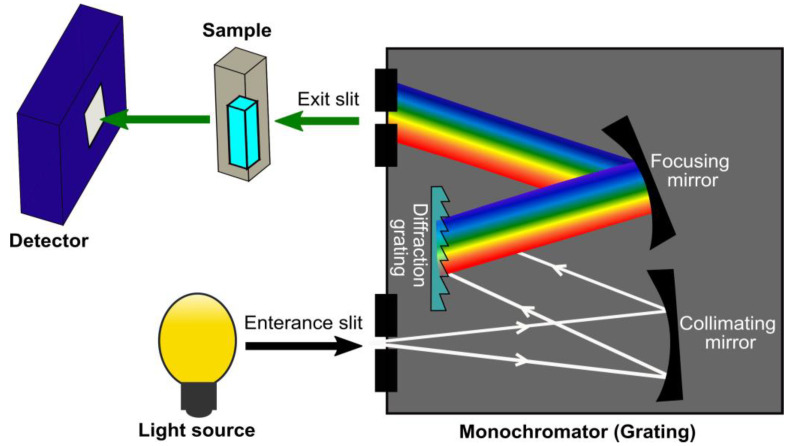
Configuration of UV-Vis/NIR spectroscopy system.

A basic NIR spectroscopy system comprises a light source, a monochromator, a sample holder, and a detector as shown in Figure 4 [48]. High-powered radiation sources such as tungsten coils or halogen lamps are typically used as the light source. The utilization of the broadband light enables scanning the absorption of the material throughout the NIR wavelength, for instance for optimisation purposes. The monochromator is an oscillating concave grating with a reflective surface, which transforms incoherent and white light into light with distinctive wavelengths across the VIS/NIR spectrum. The samples in the holder absorb radiation at specific wavelengths, while the portion of lights that are transmitted through the sample are gathered to the detector for analysis. The detector or spectrometer at the receptor side senses the signal after interaction with the material, where the detector types consist of silicon (Si), indium gallium arsenide (InGaAs), and lead sulphide (PbS) photoconductive materials [29]. A typical NIR spectroscopy consists of two dissimilar detectors, one focusing on the range of 400 to 1100 nm (Si) while the other covers between 1100 and 3300 nm (PbS). The radiation attenuations of the sample at each wavelength are plotted, assessed, and then visualized on the computer for identification or diagnosis. A crucial outcome of NIRS advancement is the awareness of the significance of correct band assignment [49]. Scanning a wider range of wavelengths may provide more significant band assignment for optimal molecular absorption wavelength determination.

**Figure 5:**
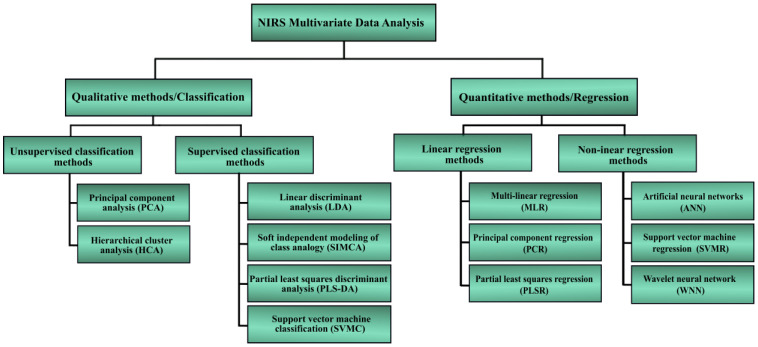
Flowchart showing commonly used methods and models in NIR analysis.

NIR spectral data analysis is categorized in two methods, which are direct and indirect. The direct analysis involves clear observation of absorption NIR spectra signature of the interest material, for instance, the absorption peak at 970 nm wavelength is clearly exhibited for a study of the presence of water in the material, or the absorption of adipose fat constituent is visibly at 930 nm wavelength [38]. The second method, which is indirect, requires further analysis using chemometric techniques. Chemometrics are used to extract pertinent data by relating the measurements through the application of mathematical or statistical methods [50,51,52]. Chemometrics correlate discrete spectral variations or inconsistencies with the material composition from the measurements, thus allowing effective detection and quantification [53]. The application of chemometrics (or multivariate data analysis) is primarily categorized into two major groups as shown in Figure 5. The first category is the qualitative discrimination or classification analysis based on supervised and unsupervised pattern identification methods, while the second group comprises multivariate methods for quantitative purposes [54,55,56]. The preference of a method is dependent on the problem, the size of the data set, the ease of implementation, and the financial viability [57]. For instance, the introduction of the NIR method in sensing applications usually requires a correlation with a valid reference method, where the gold standard is preferred. The common mathematical modelling tools for the correlation purposes include principal component regression (PCR), partial least-squares (PLS) regression, and linear regression [58,59]. Subsequently, a mathematical relationship is developed correlating the NIR spectra of the samples with the data measured from the reference method at the highest correlation value [60].

Meanwhile, the qualitative methods are normally used for the identification and differentiation of the matter of interest with other matters that possess similar characteristics, for instance, to discriminate between COVID-19 infection and the common flu or fever that show very similar symptoms as COVID-19. Principal component analyses (PCA) and soft independent modelling of class analogy (SIMCA) are the mathematical modelling examples that have been used for the discrimination and differentiation purposes [58,59]. The qualitative modelling condenses multidimensional data groups into the principal features that are derived during spectra analysis. It generates natural clusters in the data sets and explains the similarities and disparities between spectra, while the artefacts and superfluous noise are removed [61].

Recent developments in data processing strongly emphasize neural networks, machine learning, artificial intelligence, and quantum chemical calculations [62]. Up to now, other calibration models that comprise an artificial neural network (ANN), a wavelet neural network (WNN), and a support vector machine (SVM) are utilized for regression and prediction functions [56]. As a result of the advances in the chemometrics [63,64], NIRS is being applied in an extensive array of fields. The latest innovations in technology and algorithms have unlocked the path for the relevance of deep learning techniques in NIR spectral analysis. Studies have recently shown the successful deployment of deep learning in the NIRS [17,65]. It is anticipated that the awareness regarding the concept of deep networks as a data-analytical tool in NIRS will consistently grow in the near future.

### 3.2. Literature Review of Viruses Detection Using NIRS Technique

One of the ways to predict the potential of NIRS for COVID-19 detection is to study the findings from the past studies on the various types of viral detection using the similar proposed technique of NIRS. It is because most of the viruses possess somewhat similar structures and substances of having nucleocapsids (where RNA and DNA are located), envelop proteins, etc. There are groups of investigations that have reported several efforts to use NIRS in viral disease diagnosis including HIV, hepatitis, influenza, DENV, and zika (ZIKV). In this section, literature on the various types of viruses using NIRS is presented in Table 1 and an explanation including further details is provided throughout this section.

In Table 1, the use of viral samples was either direct or indirect. Similar collected samples from the patients such as blood and nasal aspirates were directly utilized in the NIRS measurements for direct types of samples. Meanwhile, the indirect samples require a few additional lab-based procedures before performing the measurements. An example of the indirect sample in Table 1 is the use of plasma in HIV-1 detection because the plasma is obtained from an anticoagulant procedure to centrifuge the collected blood [24,66]. The viral samples of heads and thoraces of the mosquitoes are also included in this study because the Aedes mosquitoes are the vectors that play an important role in pathogen transmission to humans as well as in sustaining outbreaks [67]. The direct samples are more appropriate to be used than the indirect samples, particularly for rapid and lab-free sensing unless the sensing device is incorporated with a sample-indirect-to-direct-conversion device that comes with the features of being portable, compact, and user-friendly.

Before directly testing the collected samples from infected persons and healthy volunteers on NIRS, the samples need to firstly confirm with the reference methods or the gold standard methods. Four types of reference methods were applied in Table 1, which were PCR, ELISA, immuno-chromatography, and serological tests (note that RT-qPCR is an advancement of the PCR method).

A high-performance sensing device possesses a few characteristics including a high sensitivity and a high specificity. The term sensitivity covers multiple parameters, which include the linearity, the limit of detection (LoD), and spectral signatures of the measured samples. Testing the linearity is very important for obtaining a one-to-one ratio between an independent and a dependent variable. In Table 1, the study of HIV-1 detection determined the linearity of the NIR by correlating the NIRS measurements of HIV-1 samples in different concentrations with the measurements from PCR, and the sensitivity obtained was higher, with a correlation coefficient, R2 of 0.8555. However, the prediction errors of the standard error of cross-validation (SECV) and the standard deviation (SD) were also high, which may be due to the original purpose of the device of Fruit-Tester-20 for fruits [24]. For LoD determination, a low LoD in sensing applications may provide significant differentiation between infected patients and healthy persons for high and low concentrations, respectively. The LoD for the detection of viruses using NIRS may require further study and investigation because there are none in Table 1. Nevertheless, as the clinical samples from blood, plasma, and other parts of the body were utilized, it can be assumed the LoD is within the range of detection.

In regard to the spectral signature, the absorption peaks shown in the spectrum are usually selected as the optimal wavelengths because these peaks provide important information about being sensitive to the changes in concentration of the sample. In the literature, absorption peaks due to water (or O-H vibration) at 970 nm, 1400 nm, and 1900 nm were clearly demonstrated in the studies of HIV-1, influenza, ZIKV, CHIKV, and Wolbachia [24,28,68,69]. The absorption peaks due to water for DENV and hepatitis were not seen due to the utilized range of wavelengths from 400 nm to 900 nm. The selection of wavelengths at 970 nm, 1400 nm, and 1900 nm may not be the ideal case as the optimal absorption wavelength, but the sensitivity of absorbance to the changes in concentration was still clearly demonstrated. It might be due to the reduction in water molecule components on the increases in viral concentrations. Nevertheless, these wavelengths (970 nm, 1400 nm, and 1900 nm) are suggested to be included in viral sensing for water effect elimination, especially if water is the major component in the interest samples such as saliva or nasal fluid that are composed of 90% water. The studies of DENV and hepatitis C detection in Table 1 exhibit the optimal absorption wavelengths within the visible range, with optimal wavelengths at 540 and 580 nm for DENV and 700 nm for hepatitis C. This indicates the potential of incorporating the visible wavelengths in the NIR device [70].

Possessing a high specificity is very crucial in a sensing device because it must accurately differentiate between the infected persons, infected persons due to the other viruses, and uninfected persons (e.g., between COVID-19, influenza, and normal). All viral detection studies in Table 1 applied pre-treatments and chemometric techniques for specificity determination with the obtained specificity of more than 90%. There is an exception for a study by Firdous, et al. [70], where they were able to discriminate the infected from the uninfected DENV as well as hepatitis C viruses, only from the observations using the raw spectrum. It may be due to the use of an objective lens ×10 in the NIRS system, which reveals the importance of the use of appropriate optical components in the NIRS configuration. The influenza virus was successfully separated from the uninfected in the study by Sakudo, et al., however, it (influenza infection) was redundant with the respiratory syncytial virus (RSV) in the PCA plots. Incorporating the PCA analysis with other multivariate analyses such as artificial intelligence and machine learning may be needed.

**Table 1:** A summary of studies reporting the viral infections using NIRS technology.

Detected Virus	Samples	Chemometric Analysis	Reference Methods	Wavelength Range (nm)	Limit of Detection	Sensitivity	Specificity
HIV-1 [24]	Plasma	PLS and leave-out cross-validation	PCR and ELISA	600–1000	Not applicable	Sensitive at 970 nm, R^2^ = 0.8555	-
HIV-1 [66]	Plasma	PCA and SIMCA	PCR	600–1100	Not applicable	Sensitive at 970 nm and between 1000 nm to 1100 nm	Could differentiate between HIV-1 patients and healthy
Human influenza virus [27]	Nasal aspirates	PCA and SIMCA	Immunochromatography	600–1100	Not applicable	Sensitive at 970 nm	>93%
Human influenza virus [28]	Nasal mucosal	Kruskal–Wallis test and Dunn’s	Immunochromatography	600–1100	Not applicable	Sensitive at 970 nm	-
DENV [70]	Human blood	Not applicable_	Serological test	400–900	Not applicable	Sensitive at 540 nm and 580 nm	Obvious difference between DENV, normal, and other viruses from 500–600 nm
Hepatitis C [70]	Human blood	Not applicable	Serological test	400–900	Not applicable	Sensitive at 700 nm	Obvious difference between hepatitis C, normal, and other viruses from 700–1000 nm
Wolbachia pipientis in Ae. aegypti mosquito [71]	Heads and thoraces	PLS	Not applicable	500–2350	Not applicable	Sensitive at 1400 nm and 1900 nm	>96.6% between different strains
ZIKV-infected Ae. aegypti mosquitoes [68]	Heads and thoraces	Cross-validation, Regression coefficient and PLS	RT-qPCR	350–2500	Not applicable	Sensitive at 1900 nm	>94%
ZIKV-, CHIKV, and Wolbachia-infection [69]	Heads and thoraces	PLS and PLSDA	RT-qPCR	350–2500	Not applicable	Sensitive at 1400 nm and 1900 nm	>96%

### 3.3. Potential of Coronavirus Detection Using NIRS Technology

As discussed in Section 3.2 and shown in Table 1, NIRS from the literature is already employed for the identification of diseases by various viruses including HIV, hepatitis C, influenza, DENV, ZIKV, and CHIKV. The past studies showed that NIRS for viral detection was highly influenced by water. The detection of viruses using NIR wavelengths can be based on the reduction in water molecule components on the increases in viral concentrations. The literature also showed that incorporating chemometrics analysis and machine learning, as well as implementing suitable optical components in the NIRS configuration could contribute to the performance of the NIRS.

Recent studies of COVID-19 detection using spectroscopic techniques from the years 2021 to 2022 have been domineered by the use of the mid-infrared (MIR) wavelengths, which implements the Fourier transform technique (known as FT-IR). Only one study used the ultraviolet-visible (UV-VIS) range of wavelengths as shown in Table 2. Yet, the study using near-infrared wavelengths has been still absent. In Table 2, there were two types of samples measured by the spectroscopic techniques that were the whole SARS-CoV-2 virus and ribonucleic acid (RNA). Using RNA as the sample requires the additional procedures of extraction from the whole virus that needs to be conducted by the professional health workers. Thus, RNA-based detection may not be suitable for direct detection. In Table 2, most of the sensitivity and specificity parameters measured by the spectroscopic techniques showed high percentages of more than 80%. The selection of NIR wavelengths for the detection of COVID-19 spectroscopy offers several advantages over MIR and UV, where the penetration of NIR radiation is farther into the sample than MIR radiation due to higher energy possessed by the NIR radiation. Furthermore, NIRS is less expensive that the FT-IR method. Meanwhile, compared to UV radiation, NIR radiation is considered safer, particularly for frequent detections.

**Figure 6:**
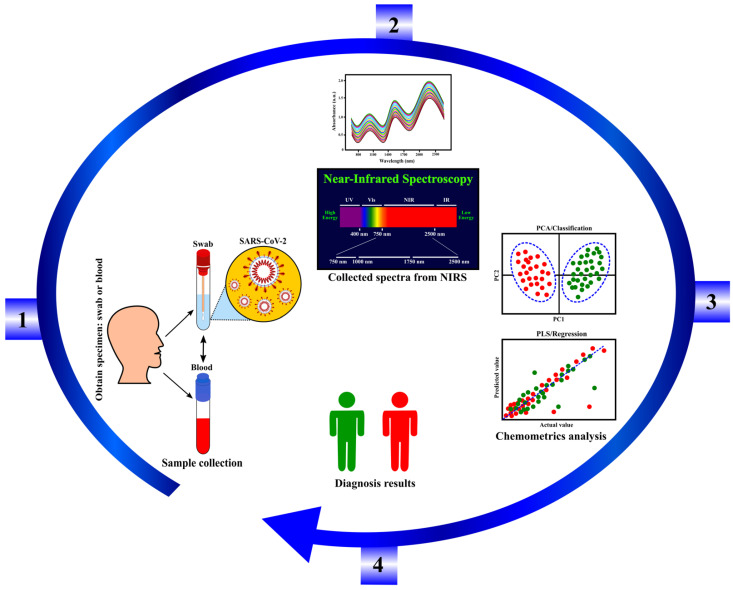
Schematic drawing of steps to detect SARS-CoV-2 using the NIRS technique.

Our perspective on current and future directions is the potential of the NIRS technique for a rapid, low-cost, compact (portable), and friendly-user device for the recognition of the SARS-CoV-2 virus. A pilot study for SARS-CoV-2 detection comprises similar steps as other viral detections in the literature (Section 3.2) above. The pilot study involves the optimization of sensitivity, the LoD, spectral signatures, and the specificity for SARS-CoV-2 virus detection, as presented in Figure 6 from steps 1 to 4. As shown in this figure, the specimen can be obtained using a swab (acquired through the nose or throat with a cotton swab) or through saliva or blood. It was reported that high viral loads are located in nasal samples as compared to the specimens obtained from the throat [72]. Both nasal and oropharynx specimens are suggested for collection to boost the accuracy and sensitivity of detection. Collecting and testing samples from various sites may always improve sensitivity and minimize the false-negative test results. Next (step 2), the spectra of samples are assembled using the NIRS instrument for wavelength selection. In this stage of the pilot study, scanning absorption over the UV-VIS range of wavelengths is also recommended. The acquired spectral signatures of SARS-CoV-2 may provide similar curves or similar absorption peaks as the other viruses in Table 1, where the NIR radiation may be more sensitive at 970 nm, 1400 nm, and 1900 nm wavelengths in the changes of SARS-CoV-2 concentration. Subsequently in step 3, the chemometrics model, such as the PCA/PLS incorporating machine learning technique, can be employed to differentiate the viral strains and fingerprint of SARS-CoV-2, in comparison to healthy and other similar symptoms of COVID-19 (such as the common flu, fevers, etc.). Testing improvements of sensitivity, specificity, etc., offered by adding the appropriate optical components in the NIRS configurations such as an attaching lens, cosine correctors, and different diameters of fiber can also be done in this stage [40,70,73]. Finally (step 4), the infected sample is pre-diagnosed and predicted.

**Figure 7:**
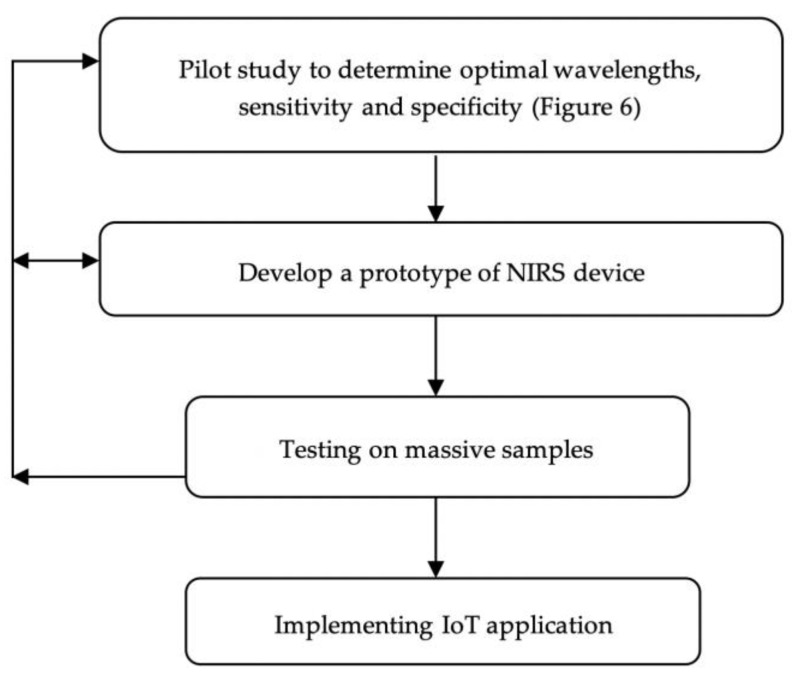
Proposed steps of an NIRS device development for COVID-19 detection.

Towards the development of a NIRS commercial device, the next step after the pilot study is the development of a prototype device using an electronics-embedded system. Various available platforms of electronics-embedded systems include Arduino, Raspberry Pi, and BeagleBone [74]. Figure 7 illustrates the proposed steps of an NIRS device development for COVID-19 detection. On the hardware part, optimization of the NIR configuration system can be done by replacing the broadband light source (in Figure 4) with multiple LEDs at the acquired optimal absorption wavelengths from the pilot study. The LED is utilized because it brings a reduction in terms of size and cost compared to the broadband light source. On the receptor side, the spectrometer that is used in the pilot study would be changed to tiny photodiodes incorporating optical components of filters and amplifiers for compact device development. Meanwhile, the software part covers the programming and coding of chemometrics analysis associating machine learning, which then links to a microcontroller in the embedded system hardware. At this stage, the sensitivity, LoD, and specificity of the developed NIR prototype device for SARS-CoV-2 detection need to be tested again.

Testing on massive samples is required for the verification and validation of accuracy, sensitivity, and specificity. The COVID-19 measurements from the developed NIRS device can be validated with a reference method such as ELISA or RT-PCR. One of the ways of validation is a determination of the correlation between the measurements from the NIRS device and the reference method by an equation model development [40]. Note that the development processes and steps of the NIRS device in Figure 7 can be back and forth if it is required for further optimization.

**Figure 8:**
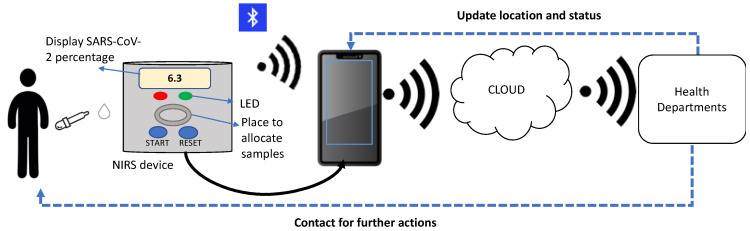
Proposed implementation of NIRS device of COVID-19 in IoT application.

Figure 8 illustrates the proposed implementation of the NIRS device in the internet of things (IoT) application. The IoT concept refers to the effective transmission of information from the sensors to the computing devices and databases via network connections such as wireless fidelity (Wi-Fi) and Bluetooth. After a user places the nasal or saliva or blood samples on an allocated space of the NIRS device in Figure 8, an LCD will show the percentage of SARS-CoV-2 virus in the sample. If the percentage falls into the infected category, then a red LED will blink. Otherwise, an LED in a green color will blink for the uninfected category.

**Table 2:** Recent studies of COVID-19 detection using spectroscopy at mid-infrared and UV-VIS wavelength.

Samples	Chemometric Analysis	Reference Methods	Wavelength Range (nm)	Limit of Detection	Sensitivity	Specificity
RNA from swab test [75]	PLS, PCA, machine learning	RT-PCR	1250–16,667 nm	10 copies/μL	97%, Sensitive at 2352 nm and at Mid IR region	98.3% between positive and negative samples
SARS-CoV-2 from saliva [76]	PCA, PLS-DA, Monte Carlo Double Cross Validation	RT-qPCR	2500–10,000 nm	Not applicable	93%	82% between positive and negative samples
Swab fluid [77]	PLS and cosine k-nearest neighbors (KNN)	RT-PCR	Mid IR	Not applicable	84–87%	64–66% between positive and negative samples
SARS-CoV-2 isolate [78]	Not applicable	Not applicable	UV-VIS	Not applicable	Sensitive at 280 nm	Not applicable
RNA from swab test [79]	Genetic Algorithm-LDA	RT-PCR	Mid IR	1582 copies/mL		89% between positive and negative samples

An installed app in a mobile phone receives the information from the NIRS device either via Bluetooth or Wi-Fi, where those Bluetooth and Wi-Fi are already equipped in the embedded electronics system. The data are then directly transferred to a database that links to health departments and other authorities. Subsequently, further fast-actions can be done by the health departments such as updating the status and possible infected locations on the app for community concerns, as well as contacting the infected COVID-19 person for treatments. This rapid detection by an NIRS device incorporating IoT applications would provide early prevention, and further could potentially reduce the number of COVID-19 cases.

## 4. Conclusions and Future Outlook

Infections by viruses are considered one of the biggest problems that threaten worldwide health. The viruses evolve with time in the human body, which boosts the possibility of new fatal cases. The annual announcement of the high number of new cases and mortalities of the respiratory viruses’ illnesses makes them a global health concern. The ongoing outbreak of COVID-19 disease troubles the human being and draws attention to the significance of the laboratory recognition of SARS-CoV-2 in order to limit its proliferation and properly treat infected persons with a critical infection. The development of the diagnosis methods for viral diseases results in controlling and a faster treatment of the infectious pathogens. The current diagnosis methods are relatively slow, costly, have the possibility of false results, and are not accessible to the community. Therefore, there is a high demand for a non-invasive, rapid, low-cost technique for recognizing SARS-CoV-2 and for a device that can provide fast-transmitting data of the community health status to the authorities for rapid prevention.

Based on the reported literature above, NIR radiation for viral detection is highly influenced by water. Incorporating the visible light sources and adding the appropriate optical components in the NIRS configuration may increase the sensitivity and specificity of the NIRS system, where the use of the light source at the NIR wavelength is to offset against the water effect. Based on the literature, it is evident that the NIR spectra of viral infections joined with chemometrics and deep learning could be a promising process for virus recognition. Furthermore, this research presented that the precision of NIRS to differentiate the changes due to virus infections was very high and this fact is very interesting. Therefore, it is anticipated in the future that NIRS technology will be employed to diagnose COVID-19 and detect structural changes in the body of infected individuals (as the head and thoraces of Aedes mosquitoes were used in the literature) instead of the antecedent methods such as PCR and ELISA. It can be concluded from this review that the NIRS technique needs an amendment to be applied for discerning the SARS-CoV-2.

The NIRS is a rapid, inexpensive, and non-destructive diagnostic approach that provides numerous benefits in diverse medical applications. The deployment of NIRS in an embedded electronics system produces a miniature and portable device. As the embedded electronics system equips with Bluetooth and Wi-Fi adapters, integrating the NIRS technology with IoT is nearly possible. Through the IoT, the information on the health status of users after using the NIR device for COVID-19 detection can be rapidly updated to the authorities such as health departments; therefore, quick prevention and precaution against the spread of COVID-19 infection among the community can be achieved.

## Data Availability

Not applicable.
